# Repurposing Auranofin, Ebselen, and PX-12 as Antimicrobial Agents Targeting the Thioredoxin System

**DOI:** 10.3389/fmicb.2018.00336

**Published:** 2018-03-05

**Authors:** Holly C. May, Jieh-Juen Yu, M. N. Guentzel, James P. Chambers, Andrew P. Cap, Bernard P. Arulanandam

**Affiliations:** ^1^South Texas Center for Emerging Infectious Disease, University of Texas at San Antonio, San Antonio, TX, United States; ^2^Center for Excellence in Infection Genomics, University of Texas at San Antonio, San Antonio, TX, United States; ^3^United States Army Institute for Surgical Research, San Antonio Military Medical Center, San Antonio, TX, United States

**Keywords:** thioredoxin, antimicrobial, antimicrobial resistance, flavoenzyme, drug target

## Abstract

As microbial resistance to drugs continues to rise at an alarming rate, finding new ways to combat pathogens is an issue of utmost importance. Development of novel and specific antimicrobial drugs is a time-consuming and expensive process. However, the re-purposing of previously tested and/or approved drugs could be a feasible way to circumvent this long and costly process. In this review, we evaluate the U.S. Food and Drug Administration tested drugs auranofin, ebselen, and PX-12 as antimicrobial agents targeting the thioredoxin system. These drugs have been shown to act on bacterial, fungal, protozoan, and helminth pathogens without significant toxicity to the host. We propose that the thioredoxin system could serve as a useful therapeutic target with broad spectrum antimicrobial activity.

## Introduction

Resistance to antimicrobial drugs is an increasingly important public health concern leading to increased mortality, morbidity, and cost of care for affected patients. Thus, discovery of novel drug targets against drug resistant pathogens is both timely and of utmost importance. However, development of new antimicrobial agents is both time-consuming and expensive, but the re-purposing of previously tested and/or approved drugs as antimicrobial therapeutic agents may be a potentially useful alternative (Ashburn and Thor, 2004). This review examines data supporting targeting of the thioredoxin system as a mechanism leading to broad spectrum antimicrobial activity against multiple species of bacteria, fungi, and eukaryotic parasites using drugs previously tested by the U.S. Food and Drug Administration (FDA).

Thioredoxins (TrxA, TrxC, Trx1, Trx2, and Trx3) are small redox proteins that possess a highly conserved active site (cys-x-x-cys) (Holmgren, 1985, 1995) referred to as the “thioredoxin motif” which consists of four α-helices and five β-sheets (Holmgren, 1995). The thioredoxin system is an efficient disulfide reduction system, consisting of two proteins: thioredoxin, the flavoenzyme thioredoxin reductase (TrxR, Trr1, and Trr2), plus the reducing agent nicotinamide adenine dinucleotide phosphate (NADPH). These ubiquitous proteins are found in all forms of life and they, along with the glutathione/glutathione reductase (GSH/GR) are responsible for maintaining a reduced cellular environment (Holmgren, 1985).

**FIGURE 1:**
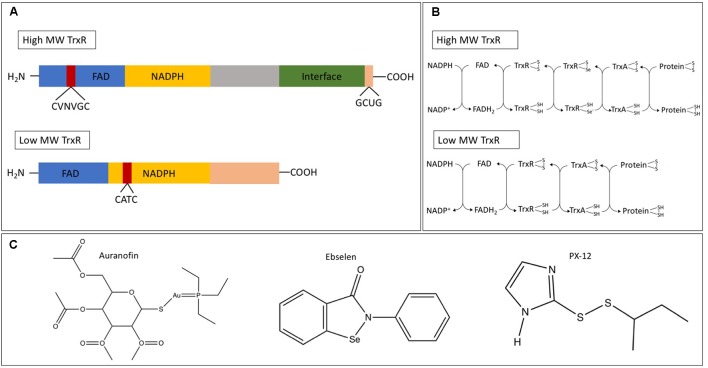
**(A)** Example of the protein structure of the high (*Homo sapiens*) and low (*Escherichia coli*) molecular weight thioredoxin reductase. Shown are the flavin-adenine dinucleotide (FAD) and nicotinamide adenine dinucleotide phosphate (NADPH) binding domains, the ‘central’ domain and the C-terminal domain that provides the dimer interface and part of the active site. **(B)** Differential electron transfer between high and low molecular weight TrxR. **(C)** Chemical structures of the thioredoxin system inhibitors auranofin, ebselen, and PX-12. Panels **(A,B)** adapted from Lu and Holmgren (2014).

Although highly conserved, there are notable differences in thioredoxin reductase. In humans and some protozoans, a high-molecular weight thioredoxin reductase (H-TrxR) is present while in bacteria, fungi, plants, and some protozoa, a low-molecular weight thioredoxin reductase (L-TrxR) is observed (McMillan et al., 2009). While both forms contain a redox active disulfide adjacent to a flavin ring, the transfer of the reducing equivalents from the flavin ring to the protein substrate have distinct mechanisms. For an in-depth analysis on these differences, refer to the review by Williams et al. (2000). **Figures 1A,B** show the differences in structure and electron transfer between H-TrxR and L-TrxR. Together, these differences allow L-TrxR containing thioredoxin systems to be a potential antimicrobial target. To that end, we review the function of thioredoxin in bacteria, fungi, protozoa, and helminths, and examine some current thioredoxin system inhibitors as potential antimicrobial agents.

### Global Functions of Thioredoxin

The thioredoxin system functions as a potent reducer of disulfide bonds. Disulfide bond reduction is initiated by a nucleophilic attack of the first cysteine of the twin cysteine motif on the disulfide of the target protein leading to formation of a mixed disulfide bond between thioredoxin and the target protein. Subsequently, the second cysteine nucleophilically attacks the mixed disulfide, forming an intramolecular disulfide bond in thioredoxin fully reducing the target protein (Roos et al., 2009). In turn, thioredoxin is reduced by thioredoxin reductase using NADPH as source of electrons (Lu and Holmgren, 2014). In all organisms studied to date, the thioredoxin system plays an important role in keeping the intracellular compartment in a reduced state which has been shown to be important in preventing protein aggregation (Holmgren, 1984; Stewart et al., 1998). Additionally, thioredoxin acts as a hydrogen donor for key enzymes involved in various cellular functions. Examples of these enzymes include ribonucleotide reductase, an essential enzyme during DNA synthesis (Reichard, 1993), and methionine sulfoxide reductase which plays an important role in protein repair (Gonzalez Porque et al., 1970; Boschi-Muller et al., 2000). Thioredoxin also is a hydrogen donor for phosphoadenosine-phosphosulfate reductase (Lillig et al., 1999; Chartron et al., 2007) which is implicated in sulfur assimilation, and required for *de novo* cysteine biosynthesis in fungi and many enteric bacteria (Gonzalez Porque et al., 1970; Russel et al., 1990).

### Drug Characteristics

Arguably the best studied inhibitor of the thioredoxin system is auranofin, a gold complex originally approved to treat rheumatoid arthritis (Bombardier et al., 1986). Although thioredoxin reductase may not be the sole target for auranofin (Thangamani et al., 2016a), the drug is believed to inhibit this enzyme by irreversibly binding thiol and selenol groups on the enzyme (Fan et al., 2014). Ebselen is an organoselenium drug that acts as an antioxidant and an anti-inflammatory agent due to its GSH peroxidase-like activity (Muller et al., 1984; Schewe, 1995), and is a potent bacterial TrxR inhibitor via its binding to the C-terminal active site cysteine residue (Lu et al., 2013). 1-Methylpropyl 2-imidazolyl disulfide (PX-12) irreversibly binds to the Cys73 cysteine residue that lies outside the conserved redox catalytic site of Trx1 (Kirkpatrick et al., 1998). Initially tested as an antitumor drug, it was not approved due to lack of efficacy in human trials; although, it exhibited low toxicity (Ramanathan et al., 2011). Like most commonly used antibiotics, the common side effects of auranofin and PX-12 include nausea, lack of appetite, diarrhea, and stomach cramps (Furst, 1983; Cunha, 2001; Ramanathan et al., 2011). Ebselen has not shown adverse effects at the recommended dose (Singh et al., 2016). Please see **Table 1** for a list of pathogens these drugs have been tested against. For chemical structures of these inhibitors, see **Figure 1C**.

**Table 1:** *In vivo* and *in vitro* studies of thioredoxin system inhibitors.

Inhibitor	Pathogen	Strain	Model	Reference
Auranofin	*S. aureus*	MW2	*C. elegans*	Fuchs et al., 2016
Auranofin	*S. aureus* (MRSA)	Sanger 252	Murine—systemic	Harbut et al., 2015
Auranofin	*S. aureus* (MRSA)	132	Murine	Aguinagalde et al., 2015
Auranofin	*C. neoformans*	Clinical isolate	*C. elegans*	Thangamani et al., 2017b
Auranofin	*T. gondii*	RH	Chicken embryo	Andrade et al., 2014
Auranofin	*G. lamblia*	WB, 106, 1279	Murine	Tejman-Yarden et al., 2013
		WB	Gerbil	
Auranofin	*T. foetus*	D1	Murine—vaginal	Hopper et al., 2016
Ebselen	*S. aureus*	USA300	Murine—skin	Thangamani et al., 2015
Ebselen	*E. coli*	ZY-1	Murine—systemic	Zou et al., 2017
Ebselen	*C. berghei*	K173	Murine	Huther et al., 1989
PX-12	*A. fumigatus*	AF-dsRed	Murine—corneal	Leal et al., 2012
Auranofin	*S. aureus*	MW2	*In vivo*	Fuchs et al., 2016
	*E. faecium*	2421		
	*K. pneumoniae*	77326		
	*A. baumannii*	ATCC 17978		
	*P. aeruginosa*	PA14		
	*Enterobacter* sp.	KCTC 2625		
	*B. subtilis*	PY 79		
	*E. faecalis*	MMH 594		
	*C. albicans*	SC5314 (CAN14)		
	*C. glabrata*	ATCC 90030		
	*Candida parapsilosis*	ATCC 22019		
	*C. tropicalis*	ATCC 13803		
	*C. neoformans*	KN99α		
Auranofin	*M. tuberculosis*	H37Ra	*In vivo*	Harbut et al., 2015
	*B. subtilis*	168, PY79		
	*S. aureus* (MRSA)	Sanger 252, TCH1516, ST-59, A7819, PA, D712		
		A5940, X18311, PC-3, HIP 5836		
	*S. aureus*	MSSA 29213		
	*E. faecium* (VRE)	VRE8 WMC, VRE 12-15-19 UCLA		
	*E. faecalis*	Belt		
	*K. pneumoniae*	1100		
	*A. baumannii*	ATCC 19606, ATCC 17978, 5075		
	*P. aeruginosa*	PA01, PA103		
Auranofin	*E. histolytica*	HM1:IMSS	*In vivo*	Debnath et al., 2012
Auranofin	*P. falciparum*	3D7	*In vivo*	Sannella et al., 2008
Auranofin	*L. infantum*	MHOM/TN/80/IPT1	*In vivo*	Ilari et al., 2012
Auranofin	*T. brucei*	449	*In vivo*	Lobanov et al., 2006
Auranofin	*E. granulosus*	Clinical isolate	*In vivo*	Bonilla et al., 2008
Auranofin	*E. coli*	ATCC 25922		
	*S. aureus*	ATCC 25923, USA300	*In vivo*	Cassetta et al., 2014
	*Staphylococcus epidermidis*	ATCC 35984, ATCC 12228		
	MRSA	Five clinical isolates		
Auranofin	*P. aeruginosa*	n/a	*In vivo*	Hokai et al., 2014
	*E. coli*	n/a		
	*S. aureus*	USA300		
	*S. cerevisiae*	USA400		
Auranofin	*C. albicans*	CA-1 to CA-13	*In vivo*	Wiederhold et al., 2017
	*C. glabrata*	CG-1 to CG-10		
	*Candida krusei*	QC		
	*C. parapsilosis*	QC, CP-1 to CP-10		
	*C. neoformans*	CN-1 to CN-3		
	*Blastomyces dermatitidis*	BD-1 to BD-3		
	*Paecilomyces variotii*	QC		
	*A. fumigatus*	AF-1 to AF-3		
	*Rhizopus oryzae*	R-1 to R-3		
	*S. apiospermum*	SA-1 to SA-7		
	*L. prolificans*	SP-1 to SP-6		
Auranofin	*S. aureus*	TCH1516	*In vivo*	Torres et al. (2016)
Auranofin	*T. gondii*	RH	*In vivo*	Andrade et al., 2014
Auranofin	*C. albicans*	SC5314	*In vivo*	Siles et al., 2013
Ebselen	*E. coli*	DHB4	*In vivo*	Lu et al. (2013)
	*H. pylori*	MSG6, MSG142, MR162		
		MRG193, NCTC11637, YS-16		
	*M. tuberculosis*	H37Rv		
		Panel 3:24		
		BTB98-310		
Ebselen	*S. aureus* (MRSA)	USA100, USA200, USA300	*In vivo*	Thangamani et al., 2015
		USA400, USA500, USA700		
		USA800, USA1000, USA1100		
		ATCC 43300, ATCC BAA-44		
	Linezolid-resistant SA	NRS119		
	Mupirocin-resistant SA	NRS 107		
	Vancomycin-resistant SA	VRS1–VRS3a, VRS3b		
		VRS4–VRS10		
	*S. epidermidis*	NRS101		
	*S. aureus*	ATCC 6538		
Ebselen	*B. subtilis*	ATCC 6633	*In vivo*	Gustafsson et al., 2016
	*S. aureus*	ATCC 29213		
	*Bacillus cereus*	ATCC 14579		
	*M. tuberculosis*	H37Rv		
Ebselen	*S. aureus*	ATCC 25923	*In vivo*	Pietka-Ottlik et al., 2008
	*Staphylococcus simulans*	103P		
	*E. coli*	ATCC 25922		
	*P. aeruginosa*	ATCC 258243		
	*K. pneumoniae*	ATCC 700603		
	*Aspergillus niger*	Filamentous		
	*C. albicans*	Yeast		
Ebselen	*E. coli*	DHB4, ZY-1, ATCC 700926	*In vivo*	Zou et al., 2017
		1139, 2219		
	*K. pneumoniae*	322		
	*A. baumannii*	H, 361		
	*P. aeruginosa*	1298, 9		
	*Enterobacter cloacae*	431, 2301		
Ebselen	*S. cerevisiae*	AH109	*In vivo*	Billack et al., 2010
Ebselen	*Aspergillus flavus*	ATCC MYA-3631	*In vivo*	Ngo et al., 2016
	*Aspergillus terreus*	ATCC MYA-3633		
	*Aspergillus nidulans*	ATCC 38163		
	*C. albicans*	ATCC 10231, 64124, MYA-2876, MYA-90819, MYA-1003, MYA-2310, ATCC 1237		
	*C. glabrata*	ATCC 2001		
	*C. krusei*	ATCC 6258		
	*C. parapsilosis*	ATCC 22019		
Ebselen	*P. falciparum*	T_9_96	*In vivo*	Huther et al., 1989
PX-12	*A. fumigatus*	AF-bp	*In vivo*	Leal et al., 2012

### Bacterial Thioredoxin

In general, bacterial thioredoxin systems are encoded by a single thioredoxin reductase, and two thioredoxin genes. The best studied system is that of *Escherichia coli* which has two thioredoxins, a higher expressed Trx1 protein (encoded by *TrxA*) with greater electron donor efficacy and a less expressed TrxC which contains two additional c-x-x-c motifs at its N-terminus (Laurent et al., 1964; Miranda-Vizuete et al., 1997). As shown by gene deletion, neither of these thioredoxin genes are required for viability in *E. coli* (Ritz et al., 2000). However, some bacteria, such as *Rhodobacter sphaeroides* (Pasternak et al., 1997), *Bacillus subtilis* (Scharf et al., 1998), and *Anacystis nidulans* (Muller and Buchanan, 1989) require a bacterial thioredoxin gene for survival. Redundant to the thioredoxin system in many bacteria is the glutaredoxin system which was initially identified as an alternative hydrogen donor for ribonucleotide reductase in an *E. coli* thioredoxin mutant (Holmgren, 1976). Simultaneous disruption of both thioredoxin and glutaredoxin systems is often lethal for bacteria (Prinz et al., 1997; Stewart et al., 1998). To date, it appears most Gram-negative bacteria contain both a thioredoxin and GSH system while most Gram-positive bacteria contain only a thioredoxin system (Lu and Holmgren, 2014). This highlights the critical role of thiol-redox homeostasis for microbial growth and further underscores antimicrobial drug potential.

Bacterial thioredoxin function and gene regulation has been reviewed previously (Zeller and Klug, 2006; Lu and Holmgren, 2014). Recent reports from animal studies have shed additional light on the important role of thioredoxin in bacterial pathogenesis. Cheng et al. (2017) propose that TrxA is essential for maintaining a highly reduced environment in the cytosol of *Listeria monocytogenes* providing a favorable environment for protein folding and subsequent activation. Furthermore, it was observed that TrxA is required for proper function of several key regulators, including (1) MogR, a DNA binding transcriptional repressor involved in flagella formation, and (2) PrfA, a member of the cAMP receptor protein (Crp) family of transcription factors which regulates several major virulence factors (ActA, LLO, and Hpt) of *Listeria*. Deletion of the TrxA gene in *Listeria* resulted in loss of motility and impairment of hemolytic activity greatly reducing virulence of this pathogen in mice.

Thioredoxin also has been shown to play a role in the virulence of *Helicobacter pylori* which uses secreted thioredoxin to reduce mucin molecules to their monomeric form decreasing mucin viscosity and allowing the organism to colonize as well as facilitating migration to the epithelial surface (Windle et al., 2000). Deletion of either the TrxA or TrxC genes in *H. pylori* impairs the organism’s ability to colonize the stomach following oral bacterial challenge (Kuhns et al., 2015).

Lin et al. (2016), while studying a *Mycobacterium tuberculosis* thioredoxin reductase (TrxB2) mutant, observed TrxB2 to be an essential thiol-reducing enzyme *in vitro*, and its deficiency lead to increased clearance of the bacterium during both the acute and chronic phases of infection. Importantly, TrxB2 depletion resulted in hyper-susceptibility to rifampin, a frontline anti-tuberculosis drug, suggesting that a thioredoxin inhibitor can be used in combination with other existing antibiotics for better control of bacterial infection. This could be of significant therapeutic value in treatment of multi-drug resistant bacteria when choice of available antibiotics is limited.

Auranofin has been tested in both Gram-positive and Gram-negative bacteria. Auranofin exhibited a lower minimum inhibitory concentration (MIC) for Gram-positive pathogens than for Gram-negative, likely due to (1) the presence of the redundant GSH system (Cassetta et al., 2014; Harbut et al., 2015; Fuchs et al., 2016), and (2) the impermeability of the drug through the outer membrane (Thangamani et al., 2016a). Using an *in vitro* enzymatic assay, Harbut et al. (2015) clearly demonstrated that auranofin inhibited both recombinant thioredoxin reductases of *M. tuberculosis* and *Staphylococcus aureus*, by reducing bacterial cellular free thiols, leading to compromised defense against oxidative stress.

Auranofin was also tested against medically relevant drug resistant pathogens collectively referred to as “ESKAPE” pathogens. This grouping includes *Enterococcus faecium*, *S. aureus*, *Klebsiella pneumoniae*, *Acinetobacter baumannii*, *Pseudomonas aeruginosa*, and *Enterobacter* species. Auranofin was found to inhibit *S. aureus*, *E. faecium*, and *A. baumannii* in bacteriostatic fashion at the MIC, but bactericidal at higher concentrations (Fuchs et al., 2016). A number of studies using *S. aureus* have recognized auranofin as a potent antibacterial compound. Using an *in vivo Caenorhabditis elegans* infection model, auranofin was shown to be protective against *S. aureus*, *Enterococcus faecalis*, and *E. faecium* via apparent targeting of the thioredoxin system (Fuchs et al., 2016). Auranofin and other related gold-compounds also were shown to exhibit significant inhibition against methicillin-resistant *S. aureus* (MRSA) (Hokai et al., 2014). Animal studies carried out using mouse models further demonstrate the potential usefulness of auranofin against cutaneous (Thangamani et al., 2016b) and implant-associated biofilm (Aguinagalde et al., 2015) infections by MRSA.

Ebselen and ebselen analogs have bactericidal effects against MRSA, *E. coli*, and *H. pylori* by blocking electron transfer to thioredoxin (Lu et al., 2013). Similar to auranofin, ebselen is highly active against bacteria lacking GSH production, e.g., *S. aureus*, *H. pylori*, *M. tuberculosis*, and *Bacillus anthracis* (Lu et al., 2013; Gustafsson et al., 2016), but less effective against Gram-negative bacteria (Pietka-Ottlik et al., 2008). Simultaneous blockage of both thioredoxin and glutaredoxin systems has been explored by Zou et al. (2017) using ebselen and silver nitrate for treatment of Gram-negative bacterial infection. These results demonstrated that silver in the presence of ebselen directly inhibited *E. coli* thioredoxin reductase, and rapidly depleted GSH resulting in elevated reactive oxygen species (ROS) production and impaired DNA synthesis leading to bacterial death. Additionally, combined treatment significantly improved survival from sepsis by *E. coli* during murine infection. In a recent study using *S. aureus* by Thangamani et al. (2015), ebselen was demonstrated to greatly reduce (1) toxin (Panton–Valentine leukocidin and α-hemolysin) production, (2) biofilm formation *in vitro*, and (3) with topical treatment of skin infection significantly reduced bacterial loads accompanied with a decreased inflammatory response.

### Fungal Thioredoxin

*Saccharomyces cerevisiae* contains both cytoplasmic and mitochondrial thioredoxin systems. Under normal growth conditions, cytoplasmic thioredoxins (Trx1 and Trx2) are both dispensable; however, simultaneous deletion of both thioredoxins slowed the rate of DNA synthesis leading to abnormal cell cycle (Muller, 1991). Thioredoxin has also been shown to function in a protective manner against ROS in *S. cerevisiae* (Kuge and Jones, 1994; Muller, 1996) as well as medically important fungi such as *Candida albicans* (da Silva Dantas et al., 2010) and *Aspergillus nidulans* (Thon et al., 2007). The yeast mitochondrial thioredoxin system, which includes a thioredoxin (Trx3) and thioredoxin reductase (Trr2), protects against oxidative stress generated during respiratory metabolism (Pedrajas et al., 1999). However, it does not appear that this mitochondrial system can substitute for the cytoplasmic thioredoxin or glutaredoxin systems, as its presence does not ameliorate the slowing of the cell cycle when Trx1 and Trx2 genes are deleted (Draculic et al., 2000). Fungal thioredoxin reductases are of the low molecular weight protein type with an overall folding structure similar to bacterial TrxR (Zhang et al., 2009). Strains lacking Trr1 gene are hypersensitive to hydrogen peroxide, temperature sensitive for growth, and have an auxotrophic requirement for methionine (Machado et al., 1997; Pearson and Merrill, 1998). Trr1 is most likely essential in *C. albicans* as a true Trr1 gene knock out could not be created; however, heterozygous strains of *C. albicans* are more sensitive to oxidative stress and exhibit a decreased pathogenicity compared to the wild-type (Zaki et al., 2012). Similarly, Trr1 was shown to be essential to *Cryptococcus neoformans* viability (Missall and Lodge, 2005).

*In vitro* auranofin inhibited medically relevant *Aspergillus fumigatus*, *Candida tropicalis*, *Candida glabrata*, and *C. albicans*, and was shown to have high efficacy against fluconazole resistant *C. albicans* as well as the hard-to-treat fungal pathogens *Scedosporium apiospermum* and *Lomentospora prolificans* (Fuchs et al., 2016; Wiederhold et al., 2017). Additionally, auranofin exhibited significant inhibition of *C. albicans* biofilm formation (Siles et al., 2013), and was shown to protect against *C. neoformans* infection in an *in vivo C. elegans* model (Thangamani et al., 2017b).

Ebselen has been shown to be effective against *S. cerevisiae* as well as fluconazole-resistant *C. albicans* (Billack et al., 2010). Additionally, ebselen analogs are effective against *C. albicans* and *Aspergillus* spp. (Pietka-Ottlik et al., 2008; Ngo et al., 2016). A recent study by Thangamani et al. (2017a), demonstrated that ebselen regulates fungal GSH and ROS production, and is a potent antifungal drug against clinically relevant isolates of both *Candida* and *Cryptococcus* in a *C. elegans* infection model.

PX-12 was tested against the filamentous fungus *A. fumigatus*, and lead to increase fungal hyphae sensitivity to both H_2_O_2_ and neutrophil-mediated killing *in vitro.* Furthermore, topical PX-12 treatment significantly enhanced neutrophil-mediated fungal killing in infected mouse corneas (Leal et al., 2012).

### Protozoa and Helminth Thioredoxin Systems

Like bacteria, protozoans contain either a thioredoxin system or a combination of thioredoxin and GSH systems. *Entamoeba histolytica*, *Trichomonas vaginalis*, and *Giardia lamblia* all possess a thioredoxin system, consisting of thioredoxin (Trx), Trx peroxidase, and an L-TrxR (Hughes et al., 2003; Coombs et al., 2004; Arias et al., 2007; Leitsch et al., 2009). *Plasmodium falciparum* possesses both GSH/GR and Trx/TrxR redox systems (Kawazu et al., 2001; Nickel et al., 2006; Kehr et al., 2010); however, loss of TrxR results in loss of viability in this pathogen (Theobald et al., 2012).

Genome sequencing of *Trypanosoma brucei* (Berriman et al., 2005), *Trypanosoma cruzi* (El-Sayed et al., 2005), and *Leishmania major* (Ivens et al., 2005) demonstrated that trypanosomatids lack genes for GSH/GR and Trx/TrxR. Instead, they rely on trypanothione, a GSH derivate, and trypanothione reductase (TryR) to maintain disulfides in a reduced form (Fairlamb et al., 1985; Fairlamb and Cerami, 1992).

TrxR also has been shown to be a virulence factor in *Toxoplasma gondii*. Deletion of *T. gondii* TrxR gene reduced the parasite’s antioxidant capacity, invasion efficiency, and proliferation significantly prolonging the survival time of mice infected with the gene-knockout parasite as compared to the wild-type strain of parasite (Xue et al., 2017). Flatworms of the class Cestoda and Trematoda possess a single flavoenzyme for both thioredoxin and GSH, and is referred to as thioredoxin GSH reductase (TGR; Ross et al., 2012). *Schistosoma mansoni* depends totally on TGR for thiol redox homeostasis. Auranofin binds to TGR leading to reduced worm burdens *in vivo* (Angelucci et al., 2010). Inhibitors of TGR will not be covered further in this review.

The antimicrobial drug metronidazole (Flagyl) depends upon reduction by a flavoenzyme to become active. Resistance to this drug has been associated with changes in amounts of flavoenzymes produced by parasites (Wassmann et al., 1999; Leitsch et al., 2010, 2011). Since metronidazole does not directly inhibit the thioredoxin system, it will not be discussed further in this review.

Auranofin has also been shown to be effective in protection against protozoa and helminthes. *E. histolytica* trophozoites were observed to be 10 times more sensitive to auranofin than to metronidazole (Debnath et al., 2012). Auranofin blocked thioredoxin reductase in multiple strains of metronidazole resistant *G. lamblia*, as well as greatly reducing the number of trophozoites in the small intestine of orally challenged newborn and adult mice and gerbils (Tejman-Yarden et al., 2013). Auranofin also has been shown to have potent antimicrobial effects both *in vivo* and *in vitro* against *T. gondii* (Andrade et al., 2014). Oral administration of auranofin for 4 days cleared *Tritrichomonas foetus* in a murine model of vaginal infection (Hopper et al., 2016). Auranofin has also been shown to inhibit the growth of *P. falciparum* (Sannella et al., 2008), the pro-mastigote stage of *Leishmania infantum* (Ilari et al., 2012), the blood stream and procyclic stages of *T. brucei* (Lobanov et al., 2006), as well as *Echinococcus granulosus* larvae (Bonilla et al., 2008).

Treatment of protozoan and helminth infections with ebselen has not been widely explored. However, Huther et al. (1989) reported that while ebselen blocked the growth of human *P. falciparum* at all stages including the invasion of erythrocytes by merozoites using highly synchronized cultures, treatment was ineffective against murine *Plasmodium berghei* in a mouse infection model.

## Conclusion

Thioredoxin is a ubiquitous redox protein found in all life forms. However, significant differences in the structure of thioredoxin reductase moieties vary among higher eukaryotes and microbes. Most importantly, all bacteria, helminths, fungi, and some protozoa contain a low molecular weight thioredoxin reductase. This differs from the high molecular weight enzyme found in mammals. Therefore, the thioredoxin system can be used as an ideal target for novel antimicrobial therapies. As drug resistance continues to grow and in the absence of introduction of new antimicrobials, the repurposing of currently available drugs may constitute a new and viable therapeutic approach.

Currently, FDA tested drugs targeting the thioredoxin system include PX-12, auranofin, and ebselen. These drugs have shown antimicrobial effects in a variety of organisms (**Table 1**). The repurposing of these previously approved drugs could allow for better treatment options, including synergistic effects with existing antimicrobial drugs, against microbial infections.

## Author Contributions

All authors have made a substantial and intellectual contribution to publish this review article. BA outlined and edited the review. HM and J-JY searched the literature and wrote the review. MG, JC, and AC provided critique and edited the review.

## Conflict of Interest Statement

The authors declare that the research was conducted in the absence of any commercial or financial relationships that could be construed as a potential conflict of interest.
